# Prognostic Performance of Inflammatory Biomarkers Based on Complete Blood Counts in COVID-19 Patients

**DOI:** 10.3390/v15091920

**Published:** 2023-09-13

**Authors:** Thilo Gambichler, Nadine Schuleit, Laura Susok, Jürgen C. Becker, Christina H. Scheel, Christian Torres-Reyes, Oliver Overheu, Anke Reinacher-Schick, Wolfgang Schmidt

**Affiliations:** 1 Department of Dermatology, Ruhr-University, 44791 Bochum, Germany; n@dineschuleit.de (N.S.); laura.susok@klinikumdo.de (L.S.); christina.scheel@klinikum-bochum.de (C.H.S.); 2 Department of Dermatology, Christian Hospital Unna, 59423 Unna, Germany; 3 Department of Dermatology, Klinikum Dortmund gGmbH, 44137 Dortmund, Germany; 4 Translational Skin Cancer Research, DKTK Partner Site Essen/Düsseldorf, West German Cancer Center, Dermatology, University Duisburg-Essen, 45122 Essen, Germany; j.becker@dkfz.heidelberg.de; 5 German Cancer Research Center (DKFZ), 69120 Heidelberg, Germany; 6 Department of Internal Medicine, Ruhr-University Bochum, 44791 Bochum, Germany; christian.torres-reyes@klinikum-bochum.de (C.T.-R.); o.overheu@klinikum-bochum.de (O.O.); w.schmidt@klinikum-bochum.de (W.S.); 7 Department of Hematology and Oncology with Palliative Care, Ruhr-University Bochum, 44791 Bochum, Germany; a.reinacher-schick@klinikum-bochum.de

**Keywords:** SARS-CoV-2, eosinophils, eosinopenia, systemic immune-inflammation, C-reactive protein, lactate dehydrogenase, ferritin, comorbidities

## Abstract

With the end of the pandemic, COVID-19 has entered an endemic phase with expected seasonal spikes. Consequently, the implementation of easily accessible prognostic biomarkers for patients with COVID-19 remains an important area of research. In this monocentric study at a German tertiary care hospital, we determined the prognostic performance of different clinical and blood-based parameters in 412 COVID-19 patients. We evaluated the neutrophil-to-lymphocyte ratio (NLR), systemic immune-inflammation index (SII), pan-immune-inflammation value (PIV), and absolute eosinopenia (AEP, 0/µL) of COVID-19 patients (*n* = 412). The Siddiqui and Mehra staging proposal, the WHO clinical progression scale, and COVID-19-associated death were used as COVID-19 outcome measures. With respect to Siddiqi and Mehra staging, patient age of older than 75 years, high C-reactive protein (CRP), absolute eosinopenia (AEP), cardiovascular comorbidities, and high ferritin were significant independent predictors for severe COVID-19. When outcome was determined according to the WHO clinical progression scale, patient age of older than 75 years, high CRP, high LDH, AEP, high neutrophil-to-lymphocyte ratio (NLR), and the presence of pulmonal comorbidities were significant independent predictors for severe COVID-19. Finally, COVID-19-associated death was predicted independently by patient age of older than 75 years, high LDH, high NLR, and AEP. Eosinopenia (< 40/µL) was observed in 74.5% of patients, and AEP in almost 45%. In conclusion, the present real-world data indicate that the NLR is superior to more complex systemic immune-inflammation biomarkers (e.g., SII and PIV) in COVID-19 prognostication. A decreased eosinophil count emerged as a potential hallmark of COVID-19 infection, whereas AEP turned out to be an accessible independent biomarker for COVID-19 severity and mortality.

## 1. Introduction

In May 2023, the World Health Organization (WHO) declared an end to the public health emergency related to coronavirus disease 2019 (COVID-19). At that time, 765 million confirmed cases and about 7 million deaths had been reported worldwide. As COVID-19 has entered an endemic phase, seasonal spikes may still inflict significant stresses on national health systems. Therefore, the implementation of prognostic markers for risk stratification, the optimization of hospitalization rates, and the monitoring of COVID-19 patients remains an important area of research [1,2,3,4,5].

Clinical features, including age and age-related comorbidities (such as lung disorders, cardiovascular disease, and diabetes mellitus) as well as elevated laboratory parameters (C-reactive protein, ferritin, D-dimer, lactate dehydrogenase, lymphocytes) have been established as consistent predictors of severe COVID-19 associated with pneumonia, subsequent immediate care unit admission, and a fatal outcome [1,2,3,6,7]. Moreover, a variety of systemic immune-inflammation biomarkers based on complete blood counts (CBCs), including the neutrophil-to-lymphocyte ratio (NLR) and systemic immune-inflammation index (SII), have shown prognostic potential in COVID-19 [6,7]. However, the relatively new and more complex pan-immune-inflammation value (PIV) has not yet been studied in COVID-19. Apart from neutrophils, lymphocytes, and platelets, the PIV also includes monocytes in its formula, which have also been shown to be involved in COVID-19 progression. Blood eosinophils have received less attention in this context than the aforementioned prognostic factors such NLR. Whereas eosinophil count was not considered a prognostic biomarker at the beginning of the pandemic [8], evidence has now accumulated showing that eosinophil count might help to differentiate COVID-19 infection from other lung conditions, and additionally serve as a predictor of disease severity [9,10,11]. Here, we conducted a comprehensive retrospective/prospective study comparing a large panel of clinical and laboratory parameters including absolute eosinophil counts and the PIV in a German cohort of patients infected with SARS-CoV-2. Data were then correlated with three different outcome measures.

## 2. Materials and Methods

### 2.1. Patients

**Figure 1:**
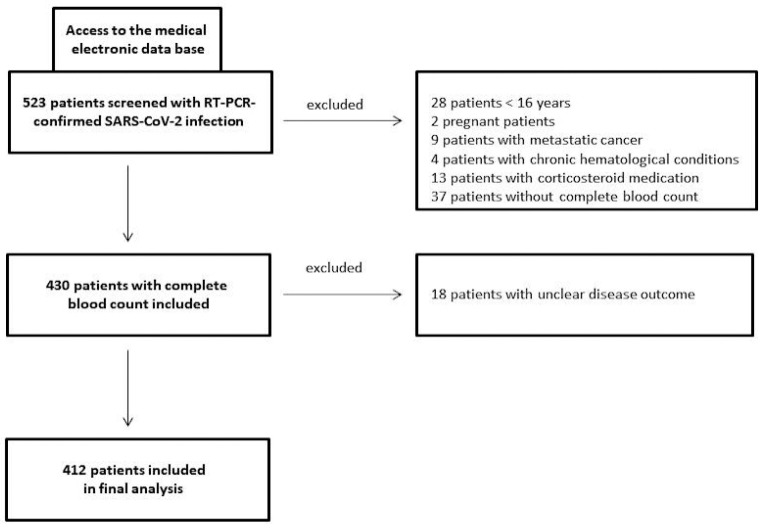
Study flow chart of COVID-19 patients investigated in a German tertiary care hospital from the beginning of 2020 to the end of 2021.

This retrospective/prospective study consecutively recruited COVID-19 patients from the beginning of 2020 to the end of 2021 at a tertiary care hospital (St. Josef) of the Ruhr-University Bochum (Bochum, Germany). Laboratory-confirmed COVID-19 patients with CBCs performed on admission were included in the study (ethics approval: #20-6953-bio). SARS-CoV-2 detection was carried out using a commercial qPCR assay on nasopharyngeal swab specimens (AllplexTM 2019-nCoV, Seegene, South Korea) according to standard protocols. Pregnant females, children (age < 16 years), those with a condition affecting laboratory parameters (including metastatic cancer or chronic haematological conditions), those who had received corticosteroid medication on admission, and those whose outcome was unknown were excluded from this investigation (Figure 1). A healthy sex/age-matched control group (*n* = 29) was also studied with respect to the CBC-based systemic immune-inflammation biomarkers PIV, SII, and NRL.

### 2.2. Data Extraction and Outcome Measures

All data were extracted from electronic medical files. These data included patient characteristics, comorbidities, length of in-patient treatment, treatment details, laboratory data, and clinical outcomes. More details are provided in Table 1 and Table 2. COVID-19 progression and the final disease outcome of COVID-19 were evaluated using three metrics. First, clinical-therapeutic staging, as proposed by Siddiqi and Mehra: I = early infection; IIA = pulmonary involvement without hypoxia; IIB = pulmonary involvement with hypoxia; and III = systemic hyperinflammation [12]. Second, the WHO clinical progression scale, which provides a measure of illness severity across a range from 0 (not infected) to 10 (dead) and groups these in stages: I = score 1–3; II = score 4 and 5; III = score 6–9; and IV = score 10 [13]. As a third measure of outcome, COVID-19-associated death was included. For statistical analysis, we dichotomized clinical-therapeutic staging by grouping stages I and IIA vs. IIB and III, and the WHO clinical progression scale by grouping stages I and II vs. III and IV. Importantly, all outcome measures were based on data easily extracted from standard electronic medical files.

### 2.3. Laboratory Tests

Parameters based on CBCs included absolute neutrophils, lymphocytes, monocytes, eosinophils, and thrombocytes. Systemic immune-inflammation biomarkers included the PIV, which was calculated from absolute values as follows: neutrophils/µL × platelets/µL × monocytes/µL by lymphocytes/µL [14,15]. The SII was calculated using the following formula: neutrophils/µL × platelets/µL by lymphocytes/µL. Using absolute blood count values, we also determined the NLR by dividing neutrophils/µL by lymphocytes/µL. Absolute eosinopenia (AEP) was defined as eosinophil count = 0/µL [10,11]. Moreover, we assessed levels of serum C-reactive protein (CRP), ferritin, and lactate dehydrogenase (LDH).

### 2.4. Statistics

For statistical analysis, MedCalc (Ostende, Belgium) software version 20.009 was used. Analysis of data distribution was performed using the D’Agostino–Pearson test. Univariable statistics included the Chi^2^ test for dichotomized data and receiver operating characteristics (ROC) analyses for continuous data [including associated criterion, area under the curve (AUC), and Youden index (optimal cut-off points of both the maximum sensitivity and specificity)]. Multivariable testing was performed using logistic regression and only included data with significance from univariate testing, specifically an AUC of ≥0.70 on ROC analysis or significance with Chi^2^ analysis. As required, variables included for testing independence did not strongly correlate with each other. Odds ratios (OR) including the 95% confidence intervals (CI) were calculated as well; *p* < 0.05 was considered statistically significant.

## 3. Results

### 3.1. Patient Characteristics and Outcome Measures

A total of 412 qPCR-confirmed COVID-19 patients were included in this study (199 females and 213 males). The median age was 58 years (range: 16–97 years, Table 1). In this cohort, 367 patients were not vaccinated (89.1%). At least two comorbidities were observed in 214 patients (51.9%). Almost all patients (389, 94.4%) were hospitalized, with a median stay of 10 days (range: 1–194). Laboratory parameters obtained on admission are detailed in Table 2. AEP was observed in 184 patients (44.7%). According to clinical-therapeutic staging as proposed by Siddiqi and Mehra, 271 patients had more severe disease (65.8%, Table 1b). By contrast, according to the WHO clinical classification scale, 108 patients were grouped as having more severe COVID-19 (26.2%).

In this cohort, 100 patients required treatment in the intensive care unit (ICU) with a median stay of 10 days (24.3%, range 1–85). Ventilation support of any kind was observed in 272 patients (66%). COVID-19-associated death was observed in 55 patients (13.3%).

### 3.2. Univariable Analysis

A comparison with healthy controls revealed that the NLR (*p* < 0.0001) and SII (*p* = 0.0002), but not the PIV (*p* = 0.47), were significantly higher in COVID-19 patients (Table 3). Moreover, PIV failed to reach an AUC ≥ 0.70 on ROC analysis for each of the three outcome measures studied. Univariable analyses are shown in Table 3, including all significant parameters for Chi² test analysis and significant parameters for ROC analysis with an AUC ≥ 0.70.

Significant predictors for severe disease (stages IIB and III) according to clinical-therapeutic staging as proposed by Siddiqi and Mehra included high ferritin, LDH, CRP, a patient age of above 75 years, AEP, and comorbidities, including cardiovascular diseases, lung diseases, diabetes mellitus, and the presence of two or more comorbidities (Table 3). Significant predictors for severe disease (classes III and IV) according to the WHO clinical classification scale included high LDH, CRP, age, AEP, and NLR, and comorbidities, including cardiovascular diseases, lung diseases, and obesity, and the presence of two or more comorbidities (Table 3). COVID-19-associated death was significantly associated with AEP, high LDH and CRP, age, systemic immune-inflammation biomarkers such as NLR and SII, and comorbidities, including cardiovascular diseases, lung diseases, and diabetes mellitus, and the presence of two or more comorbidities.

### 3.3. Multivariable Analyses

Using clinical-therapeutic staging as proposed by Siddiqi and Mehra for multivariate analysis, the following parameters emerged as independent predictors of severe disease (stages IIB and III): age greater than 75 years (OR 2.3, 95% CI 1.1 to 4.7, *p* = 0.021), high CRP (OR 4.5, 95% CI 2.2 to 9, *p* < 0.0001), AEP (OR 4.4, 95% CI 2.4 to 7.6, *p* < 0.0001), absence of cardiovascular comorbidities (OR 0.43, 95% CI 0.20 to 0.88, *p* = 0.022), and high ferritin (OR 1.0006, 95% CI 1.0001 to 1.0012, *p* = 0.026, Table 4).

When the WHO clinical classification system was used as an outcome measure for severe disease (classes III and IV), age greater than 75 years (OR 2.3, 95% CI 1.2 to 4.4, *p* = 0.011), high CRP (OR 4.4, 95% CI 2.4 to 7.6, *p* < 0.0001), high LDH (OR 2.7, 95% CI 1.4 to 5.0, *p* = 0.027), AEP (OR 3.2, 95% CI 1.8 to 5.6, *p* = 0.0001), high NLR (OR 2.5, 95% CI 1.3 to 4.9, *p* = 0.006), and the presence of pulmonal comorbidities (OR 2.3, 95% CI 1.1 to 4.6, *p* = 0.013) emerged as independent predictors. COVID-19-associated death was independently predicted by age greater than 75 years (OR 8.3, 95% CI 3.5 to 19.8, *p* < 0.0001), high LDH (OR 4.2, 95% CI 1.8 to 10, *p* = 0.0012), high NLR (OR 2.8, 95% CI 2.1.1 to 7.4, *p* = 0.035), and AEP (OR 2.6, 95% CI 1.2 to 5.7, *p* = 0.017).

## 4. Discussion

Among other findings, COVID-19 is characterized by the infiltration of infected tissues by macrophages/monocytes, as well as lymphopenia and neutrophilia in the peripheral blood of patients. Innate immune cells are activated by viral components, resulting in enhanced interferon production. Moreover, the release of endothelial cytokines that increase capillary permeability leads to the activation of platelets, enhanced coagulation, reduced fibrinolysis, and overactivation of the complement system [1,3,16]. Together, these known pathomechanisms provide the rationale to include lymphocyte, neutrophil, monocyte, and platelet counts to assess complete-blood-count-based biomarkers to predict COVID-19 outcome [17]. The NLR, determined at the time of admission to the hospital, has been frequently used as a prognostic biomarker and was included in the present study as a comparator to more complex markers such as the PIV, which has not yet been studied in COVID-19 patients [17,18,19]. Indeed, our data confirm results of previous studies showing that the baseline NLR is an independent predictor of COVID-19-associated death and severity, as classified by the WHO. In comparison to the NLR, the SII, including not only neutrophils and lymphocytes, but also platelets, was more infrequently studied in COVID-19 patients [17]. Ballaz and Fors recently suggested that a complex interaction between inflammation and haemostasis may be the reason for the modest performance of SII in the prediction of severe COVID-19 [18]. Nonetheless, SII may serve as an indicator of the inflammation levels resulting from COVID-19, which could ultimately predict death [18]. In the present study, an SII greater than 1196 was significantly associated with COVID-19-associated death but failed to independently predict this outcome as assessed by multivariate analysis. In this regard, the NLR appears to outperform SII with respect to assessing COVID-19 mortality. In line with our findings, Karimi et al. showed, in their review on novel prognostic inflammatory biomarkers for COVID-19, that most previously studied parameters are able to predict COVID-19 prognosis; however, the NLR appears to be the most robust biomarker [17,18]. Since macrophages/monocytes are also involved in the progression to severe disease, we also included the PIV in our analysis, which incorporates monocyte counts [16]. However, the PIV did not turn out to be a significant predictor of COVID-19 outcome for any of the measures used in our study. Interestingly, it appears that PIV may be a more suitable biomarker for cancer patients [14,15].

The role of biomarkers, including age, CRP, ferritin, LDH, and comorbidities, have been extensively discussed in previous studies [1,2,3]. We have shown that the aforementioned parameters are more or less strong independent predictors for three outcome measures: COVID-19-associated death, disease severity according to clinical-therapeutic staging, as proposed by Siddiqi and Mehra staging, and the WHO clinical progression scale [12,13]. In our study population, almost 45% of patients presented with AEP on admission, and 74.5% patients had some degree of eosinopenia. Indeed, Soni reported that eosinopenia (<50/µL) on admission is a reliable and convenient early diagnostic biomarker for COVID-19 infection (sensitivity 80.7%, specificity 100%), aiding in the early identification, triaging, and isolation of patients until qPCR results are available [10]. However, eosinopenia was not significant as a prognostic predictor in that study [10]. Hence, we aimed to investigate a more stringent scenario using AEP as a dichotomous variable. In the literature, we found only two studies investigating AEP in the setting of COVID-19 [9,11]. Cazzaniga et al. studied 107 patients with COVID-19-associated pneumonia and observed that AEP was associated with clinical outcomes such as mortality [9]. Ito et al. observed that AEP was a significant risk factor for ICU admission in an Asian population (*n* = 125) [11]. However, they did not detect a significant correlation with 30-day mortality [11]. By investigating a larger cohort of patients, we have shown that AEP is an easily obtained and inexpensive hematologic biomarker with good prognostic accuracy for severe and fatal COVID-19. Indeed, eosinophils reportedly have an antiviral effect, including the rapid capture and inactivation of viruses and viral blood load [11,20].

Inherent to the study design, our mixed retrospective/prospective analysis was limited by data collected in a real-world clinical environment, potentially leading to information bias. Clinical judgments by treating clinicians directed data collection, possibly resulting in missing data and incomplete analyses. Given the overwhelming workload and pressure stressing the healthcare system during the first COVID-19 infection waves, some parameters of the hospital stay were not collected, leading, in some cases, to a lack of detail. Thus, we analysed all biomarkers only at the time of hospital admission and not longitudinally. Moreover, we mainly included patients hospitalized during the first infection waves in Germany, and as such our results cannot necessarily be extrapolated to other patient populations analysed during later waves. Finally, co-infection with other pathogens and comorbidities may also be confounding factors for this study with respect to COVID-19 death rates. However, the strengths of the present study include a reasonable sample size and the fact that AEP was correlated with three different outcome measures.

In conclusion, a further assessment of biomarkers that predict severe COVID-19 remains not only of vital importance with respect to mortality, but also for increased risk for the development of long COVID-19 [21,22]. As a prognostic marker for the disease severity of COVID-19, the NLR is superior to more complex biomarkers such as SII and PIV. More importantly, a decreased eosinophil count appears to be a hallmark of COVID-19 infection, with AEP emerging as a potential independent predictor for COVID-19 severity and mortality. Hence, further studies are needed, notably on larger, prospective cohorts, and probably as part of a multi-centre and multi-marker approach in order to further evaluate the prognostic power of easily available and inexpensive CBC-based parameters such as AEP in patients with COVID-19.

## Figures and Tables

**Table 1:** Descriptive clinical baseline data of COVID-19 patients (*n* = 412) treated in a German tertiary care hospital (**a**). Comorbidities and course of COVID-19 are shown in part (**b**).

(a)		(b)	
Parameter	Data	Parameter	Data
**Sex** Female/male	199/213 (48.3%/51.7%)	**Obesity** no/yes	324/88 (78.6%/21.4%)
**Median age** (years)	58 (16–97)	**Diabetes mellitus** no/yes	314/97 (76.4%/23.6%)
**Body mass index** (kg/m^2^)	27.7 (17.3–50.5)	**Smoking** no/yes	359/53 (87.1%/12.9%)
**Vaccination status** No vaccination 1st vaccination 2nd vaccination 1st booster 2nd booster	367 (89.1%) 19 (4.6%) 23 (5.6%) 3 (0.7%) 0 (0%)	**Lung diseases** no/yes	338/74 (82%/18%)
**Recovery rate** no/yes	409/3 (99.3%/0.7%)	**Cardiovascular diseases** no/yes	189/223 (45.9%/54.1%)
**Median Ct-value** (S-gene) (E-gene) (RdRP-gene) (N-gene)	22 (7–38) 23 (10–37) 24 (7–39) 25 (11–287)	**Neuropsychiatric diseases** no/yes	328/84 (79.6%/20.8%)
**Fever (≥38 °C)** no/yes	319/79 (80.2%/19.8%)	**At least two comorbidities** no/yes	198/214 (48.1%/51.9%)
**Dysnosomie** no/yes	326/86 (79.1%/20.9%)	**Staging by Siddiqi and Mehra** Stage I Stage IIA Stage IIB Stage III	89 (21.6%) 52 (12.6%) 223 (54.1%) 49 (11.9%)
**Breathing rate** median	19 (8–50)	**WHO clinical progression scale** I II III IV	93 (22.6%) 211 (51.2%) 54 (13.1%) 55 (13.3%)
**Oxygen saturation** median percentage	97 (40–100)	**COVID-19 pneumonia** no/yes	104/307 (25.3%/74.7%)
**Days with symptoms before admission** median	6 (1–22)	**Intensive care unit (ICU)** no/yes median days on ICU	312/100 (75.7%/24.3%) 9.5 (1–85)
**In-patient treatment** no/yes	23/389 (5.6%/94.4%)	**Deceased with COVID-19** no/yes	357/55 (86.7%/13.3%)
**Duration of in-patient treatment** median	10 (1–194)	**Duration of in-patient treatment** median	10 (1–194)
		**Breathing support** no oxygen via nasal canula high-flow oxygen, non-invasive ventilation intubation ECMO	140 (34%) 179 (43.4%) 56 (13.6%) 26 (6.3% 11 (2.6%)
		**Anti-COVID-19 therapy** no dexamethasone remdesivir dexamethasone/remdesivir miscellaneous	216 (52.4%) 97 (23.5%) 31 (7.5%) 48 (11.7%) 20 (4.9%)

**Table 2:** Baseline laboratory data including systemic immune-inflammation markers of COVID-19 patients treated in a German tertiary care hospital.

Parameter	Data
**C-reactive protein (mg/L)** median (range)	36.3 (1–558)
**Lactate dehydrogenase (U/L)** median (range)	261.5 (86–1156)
**Ferritin (ng/mL)** median (range)	409 (5–10,627)
**Neutrophils (/µL)** median (range)	3955 (900–18,200)
**Lymphocytes (/µL)** median (range)	1090 (240–5090)
**Monocytes (/µL)** median (range)	440 (70–7300)
**Eosinophils (/µL)** median (range) **Eosinopenia (<40/µL)** no/yes **Absolute eosinopenia (0/µL)** no/yes	10 (0–480) 105/307 (25.5%/74.5%) 228/184 (55.3%/44.7%)
**Thrombocytes (/µL)** median (range)	189,000 (24,000–784,000)
**Neutrophil-to-lymphocyte ratio** median (range) healthy controls	3.7 (0.55–72.6) 1.9 (0.9–11.6) *p* < 0.0001
**Systemic immune-inflammation index** median (range) healthy controls	688 (39.7–14,661) 425 (39.3–5946) *p* = 0.0002
**Pan-immune-inflammation value** median (range) healthy controls	288 (16.8–24,338) 275 (81–1621) *p* = 0.47

**Table 3:** Univariable analysis including receiver operating curves (ROC) and Chi² tests in order to determine significant prognostic biomarkers for the outcome of patients with COVID-19. We exclusively included parameters revealing a significant AUC ≥ 0.70 on ROC analysis or a statistically significant result on Chi^2^ test.

Parameter	Prognostic for Class IIB and III (Siddiqi and Mehra) [12]	Prognostic for Class III and IV (WHO) [13]	Prognostic for COVID-19 Death
Ferritin	AUC 0.77, *p* < 0.0001 Criterion: >465, Youden index: 0.41	-	-
LDH	AUC 0.81, *p* < 0.0001 Criterion: >239, Youden index: 0.50	AUC 0.78, *p* < 0.0001 Criterion: >371, Youden index: 0.41	AUC 0.78, *p* < 0.0001 Criterion: >339, Youden index: 0.45
C-reactive protein	AUC 0.85, *p* < 0.0001 Criterion: >26, Youden index: 0.54	AUC 0.81, *p* < 0.0001 Criterion: >83, Youden index: 0.47	AUC 0.79, *p* < 0.0001 Criterion: >47, Youden index: 0.46
Age > 75 years	*p* < 0.0001	*p* < 0.0001	*p* < 0.0001
Diabetes	*p* = 0.014	-	*p* < 0.0001
Obesity	-	*p* = 0.0047	-
Cardiovascular diseases	*p* < 0.0001	*p* = 0.0004	*p* < 0.0001
Lung diseases	*p* = 0.006	*p* < 0.0001	*p* = 0.0073
Two or more comorbidities	*p* < 0.0001	*p* < 0.0001	*p* < 0.0001
Absolute eosinopenia	*p* < 0.0001	*p* < 0.0001	*p* < 0.0001
NLR	-	AUC 0.72, *p* < 0.0001 Criterion: >5.4, Youden index: 0.32	AUC 0.74, *p* < 0.0001 Criterion: >7.4, Youden index: 0.47
SII	-	-	AUC 0.70, *p* < 0.0001 Criterion: >1196, Youden index: 0.37

**Table 4:** Multivariable analyses (logistic regression models) included dependent variables such as COVID-19-associated death and class 2 and 3 classifications according to the Siddiqi and Mehra classification and WHO clinical progression scale [12,13]. Independent variables were included in the model if there was a significant AUC ≥ 0.70 on ROC analysis or a statistically significant result on Chi² testing for dichotomous variables.

Parameter	Prognostic for Class IIB and III (Siddiqi and Mehra) [12]	Prognostic for Class III and IV (WHO) [13]	Prognostic for COVID-19 Death
Ferritin	OR 1.0006, 95% CI 1.0001 to 1.0012, *p* = 0.026	-	-
LDH	-	OR 2.7, 95% CI 1.4 to 5.0, *p* = 0.027	OR 4.2, 95% CI 1.8 to 10, *p* = 0.0012
C-reactive protein	OR 4.5, 95% CI 2.2 to 9, *p* < 0.0001	OR 4.4, 95% CI 2.4 to 7.6, *p* < 0.0001	-
Age	OR 2.3, 95% CI 1.1 to 4.7, *p* = 0.021	OR 2.3, 95% CI 1.2 to 4.4, *p* = 0.011	OR 8.3, 95% CI 3.5 to 19.8, *p* < 0.0001
Absence of cardiovascular diseases	OR 0.43, 95% CI 0.20 to 0.88, *p* = 0.022	-	-
Lung diseases	-	OR 2.3, 95% CI 1.1 to 4.6, *p* = 0.013	-
Absolute eosinopenia	OR 4.4, 95% CI 2.4 to 7.6, *p* < 0.0001	OR 3.2, 95% CI 1.8 to 5.6, *p* = 0.0001	OR 2.6, 95% CI 1.2 to 5.7, *p* = 0.017
NLR	-	OR 2.5, 95% CI 1.3 to 4.9, *p* = 0.006	OR 2.8, 95% CI 2.1.1 to 7.4, *p* = 0.035

## Data Availability

The data that support the findings of this study are available from the corresponding author upon reasonable request.
